# Purification and fractionation of phycobiliproteins from *Arthrospira platensis* and *Corallina officinalis* with evaluating their biological activities

**DOI:** 10.1038/s41598-023-41001-y

**Published:** 2023-08-31

**Authors:** Mona M. Ismail, Esmail M. El-Fakharany, Ghada E. Hegazy

**Affiliations:** 1 National Institute of Oceanography & Fisheries, NIOF, Cairo, Egypt; 2 Protein Research Department, Genetic Engineering & Biotechnology Research Institute (GEBRI), City of Scientific Research & Technological Applications, Alexandria, Egypt; 3 Bioprocess Development Department, Genetic Engineering & Biotechnology Research Institute (GEBRI), City of Scientific Research & Technological Applications, Alexandria, Egypt

**Keywords:** Biotechnology, Microbiology

## Abstract

Phycobiliproteins (PBPs) are a class of water-soluble pigments with a variety of biological functions that are present in red macroalgae and cyanobacterial species. The crude forms of phycocyanin (C-PC) from the blue green alga *Arthrospira platensis* and allophycocyanin (APC) from the red macroalga *Corallina officinalis* were extracted and purified by ammonium sulphate precipitation, anion exchange chromatography, and size exclusion chromatography methods, respectively. The obtained C-PC and APC from *A. platensis* and *C. officinalis* were 0.31 mg/mL and 0.08 mg/mL, respectively, with molecular masses of “17.0 KDa and 19.0 KDa” and “15.0 KDa and 17.0 KDa” corresponding to α and β subunits, respectively. FT-IR was used to characterize the purified APC and C-PC in order to look into their structures. Highly purified extracts (A620/A280 > 4.0) were obtained from subtractions’ PC3 and PC4 that were tested for their biological activities. APC and C-PC crude extracts plus their fractions exhibited potent anti-oxidant in different ratios by using three techniques. PC1 showed high anti-inflammatory (75.99 and 74.55%) and anti-arthritic (78.89 and 76.92%) activities for *C. officinalis* and *A. platensis*, respectively compared with standard drugs (72.02 and 71.5%). The methanolic and water extracts of both species showed greater antibacterial efficacy against Gram +ve than Gram −ve marine bacteria. Our study shed light on the potential medical uses of C-PC and APC extracted from the tested species as natural substances in a variety of foods and drugs. Further investigations are required to explore the diverse chemical natures of distinct PBPs from different cyanobacteria and red algae because their amino acid sequences vary among different algal species.

## Introduction

Cyanophyte, Cryptophyte, Cyanelle, and Rhodophyte species have phycobilisomes (PBSs) that act as antennae of the photosynthetic pigment apparatus. Phycobilisomes contain several phycobiliproteins (PBPs) which are a category of proteinaceous accessory pigments that allow these algal species to harvest light energy outside the wavelengths absorbed by chlorophyll and carotenoids, and are responsible for about 50% of light capture from cyanobacteria and red algae^1^. These highly coloured water soluble proteins contribute 30–50% of the total light-harvesting capacity of these biotas by absorbing light in the visible range of 450–650 nm, where chlorophyll absorbs poorly in this range. They then transfer the energy to the protein chlorophyll complexes of photosystem 2 in the photosynthetic lamellae^2^.

There are over ten different known PBPs, which can be classified into four groups based on the wavelength and the presence of different chromophores: phycoerythrins (PEs) have a peak at 545–566 nm; phycoerythrocyanins at 480–580 nm; phycocyanins (PCs) at 569–645 nm; and allophycocyanins (APCs) at 540–671 nm^3^. The abundance of PBPs is very high (approximately 40–60% of the total protein content and 20% of the dry weight of cyanobacteria)^4^. PBPs (PE, PC, and APC) vary depending on their taxonomic position and culture conditions^2^, The phycobiliproteins are made up of dissimilar α and β polypeptide subunits^5^. Cyanobacterial and red algal species are the main algae used for the commercial production of phycobiliproteins, which are used as dyes, fluorescent labels, and diagnostic tools^6^. The phycobiliproteins extraction method includes cell rupture in order to release the proteins from inside algal cell to outside. While cyanobacteria’s cell walls are incredibly resilient, those of cryptophytes are very susceptible to disruption^7^. In particular, extraction of phycocyanin is challenging due to thick cell walls and high levels of contaminants^8^. Due to their ant-oxidative, anti-tumor, and photosensitizing properties as well as their utility as fluorescent markers, PBPs have recently attracted a lot of interest in the biotechnology fields of food and medicine^3,9^. The pharmaceutical industry is more interested in PBPs research for medicinal applications. Based on the report from Future Market Insights, the PBPs market were worth USD 112.3 million in 2018 and are anticipated to double in value by 2028^10^. Both C-PC and APC have been described as strong anti-oxidant agent against free radicals, might be related to their protein moieties that are crucial for the free radical scavenging process^11^. Especially, C-PC has been used as a natural protein in food and biomedical research due to its hepatoprotective, anti-oxidative, free radical scavenger, anti-inflammatory, anti-arthritic, anti-tumor activities and fluorescent labelling in biomedical research^12^. Commercially, C-PC is produced using strains of cyanobacteria like *A. platensis*^13^. According to several studies, *A. platensis* generates PC as its primary pigment in addition to APC and trace concentrations of PE^14^. Economical applications of C-PC are mainly dependent on its purity, which is usually polluted with other photosynthetic proteins, particularly APC^12^. Furthermore, APCs are frequently employed as fluorescent protein probes in biochemical procedures, especially flow cytometry^15,16^. APCs have many biotechnological applications, including anti-oxidant^17^, and anti-virus^18^. Despite the fact that APC is a helpful protein, its use is somewhat constrained by the difficulties of purifying large quantities of the protein. Due to the low concentration of APC in cyanobacteria and macroalgae, which makes its separation and purification in considerable amounts challenging work, we place special emphasis on purifying APC from *C. officinalis*. The objectives of this study were aimed to identify the dominant phycobiliproteins in two different algae species *Corallina officinalis* from Rhodophyta (APC), and *Arthrospira platensis* from Cyanophyta (C-PC). In addition to evaluate the anti-oxidant, anti-inflammatory, anti-arthritic, and anti-bacterial activities of each fraction in vitro.

## Result and discussion

### Purification and characterization of phycobiliproteins

**Figure 1:**
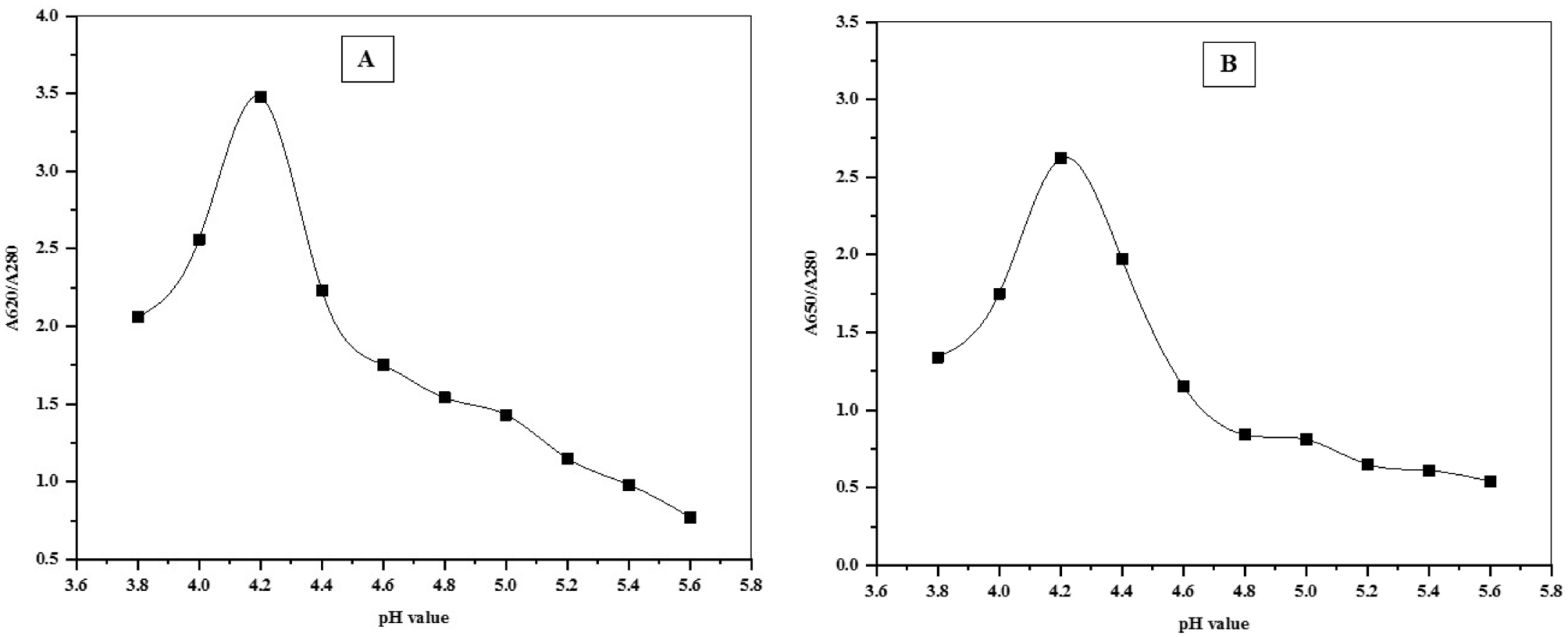
Elution profile curve of C-PC from *A. platensis* (**A**) and APC from *C. officinalis* (**B**) by anion-exchange chromatography using acetate buffer in the pH range of 3.8–5.6.

**Figure 2:**
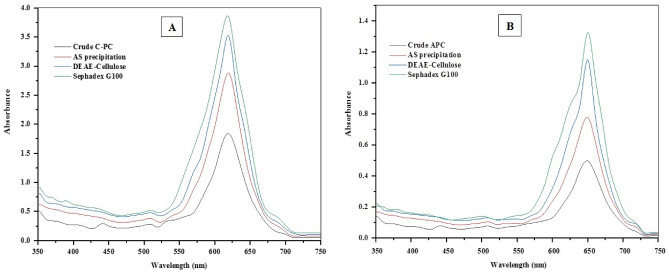
Absorption spectra of phycobiliproteins at each purification step. (**A**) UV–visible absorption spectrum of C-phycocyanin from *A. platensis* during separation and purification steps. (**B**) UV–visible absorption spectrum of allophycocyanin from *C. officinalis* during separation and purification steps.

**Figure 3:**
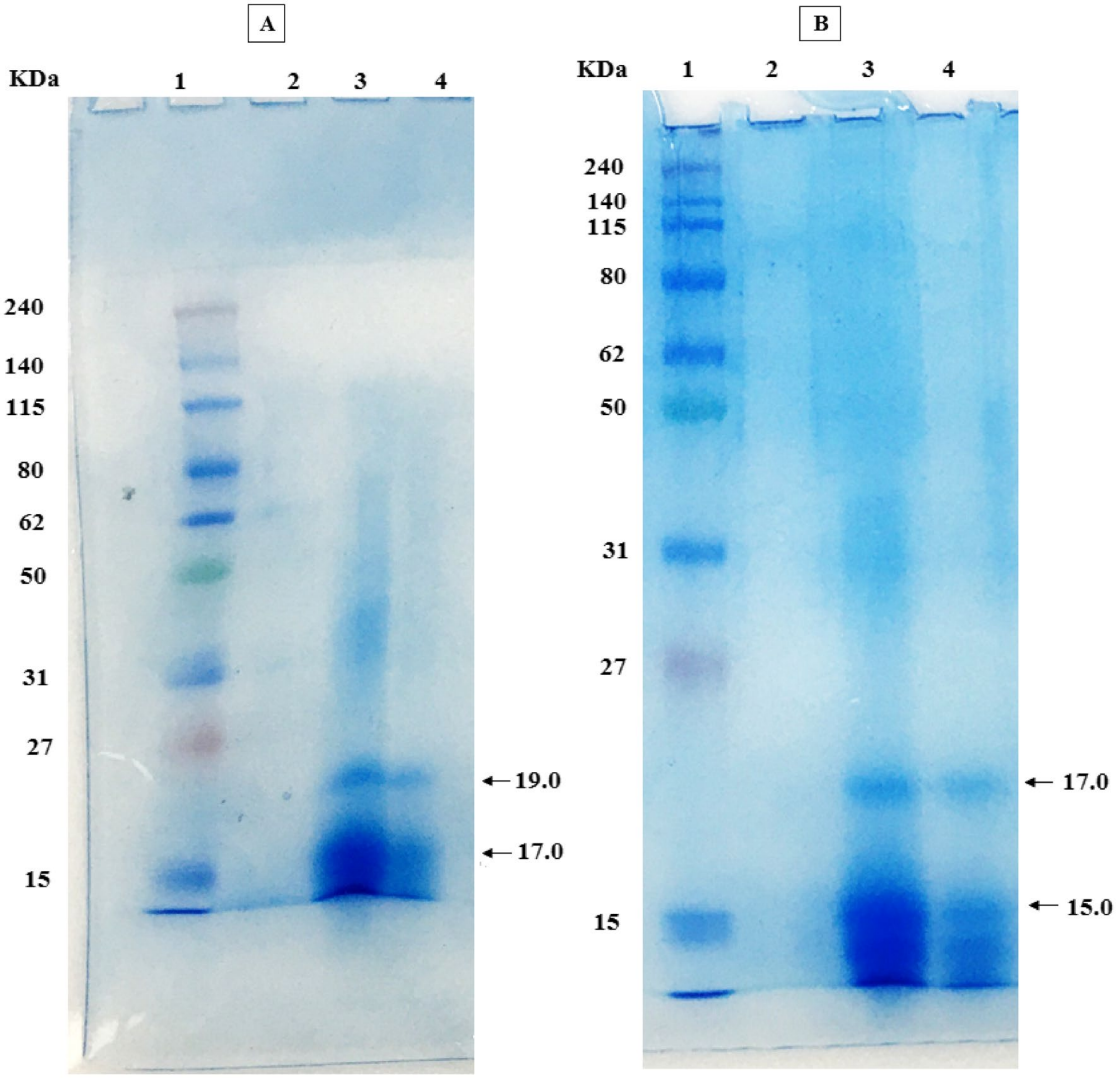
12% SDS-PAGE analysis of the purified phycobiliproteins during purification steps. (**A**) SDS-PAGE analysis of the purified phycocyanin from *A. Platensis*; lane 1 is protein molecular weight marker, lane 2 is outlet from CM Sephadex column, lane 3 is phycocyanin eluted from CM Sephadex column and lane 4 is the purified phycocyanin eluted from CM Sephadex column (**B**) SDS-PAGE analysis of the purified allophycocyanin *C. officinalis* Lane 1 is protein molecular weight marker, lane 2 is outlet from CM Sephadex column, lane 3 is allophycocyanin eluted from CM Sephadex column and lane 4 is the purified allophycocyanin eluted from CM Sephadex column.

Purification of phycobiliproteins from *A. platensis* and *C. officinalis* was optimized for its maximum recovery and purity level, which indicate the purification degree for each protein (Table 1). Both phycobiliproteins were purified through three successive purification steps, including ammonium sulfate precipitation, anion exchange chromatography using DEAE cellulose columns and size exclusion chromatography using Sephadex G100 columns. The purity index of each protein was found to be increased from 0.87 up to 5.64 for C-PC from *A. platensis* and increased from 0.49 up to 5.51 for APC from *C. officinalis* (Table 1). Precipitation of phycobiliproteins crude with 65% saturation of ammonium sulfate resulted in a purity of 2.56 and 2.23 for the obtained precipitates from *A. platensis* and *C. officinalis*, respectively, with a slight increase in the purity index after dialysis for both phycobiliproteins (2.91 and 2.38). During the chromatographic purification, both C-PC and APC were separated efficiently by anion exchange chromatography (DEAE-Cellulose) with a gradient of pH from 3.8 to 5.6 to obtain one main eluting peak containing phycobiliproteins with purity ratio reached 3.48 and 2.62 for the purified C-PC and APC, respectively (Fig. 1). After anion exchange chromatography step, the total recoveries of *A. platensis* C-PC and *C. officinalis* APC reached 51.28% and 56.89% from the starting crude extracts, equating to 2.66 and 0.787 mg/mL, respectively (Table 1). According to the absorption spectra showed maximum absorption at 620 nm and 650 nm for the purified phycobiliproteins from *A. platensis* and *C. officinalis*, respectively. These results indicate that the phycobiliprotein types are of C-PC and APC nature from *A. platensis* and *C. officinalis*, respectively (Fig. 2A and B). The purification of both C-PC and APC was found to be enhanced after each purification step (Fig. 2). From crude extract to purified phycobiliproteins, the purity was increased by almost six times, which showed the efficiency of the method to obtain high purity C-PC and APC. In addition, during size exclusion chromatographic separation, both C-PC and APC proteins showed a maximum purity of 5.64 and 5.51, respectively. The total recoveries of C-PC and APC after size exclusion chromatography reached 41.8% and 40.72%, equating to 3.20 and 0.85 mg/mL, respectively (Table 1). The SDS-PAGE results of the purified C-PC from *A. platensis* revealed two bands of 17.0 KDa and 19.0 KDa corresponding to α and β subunits, respectively (Fig. 3A). While the purified APC from *C. officinalis* revealed two bands of 15.0 KDa and 17.0 KDa corresponding to α and β subunits, respectively (Fig. 3B).

**Table 1:** Purity and recovery yield of C-PC from *Ar. platensis* and APC from *C. officinalis* during purification steps.

Purification step	*Arthrospira platensis* C-PC				*Corallina officinalis* APC			
	Total volume (mL)	Total C-PC (mg/ml)	Purity index (A620/A280)	Recovery (%)	Total volume (mL)	Total PE (mg/ml)	purity index (A650/A280)	Recovery (%)
Crude extract	100	0.31	0.87	100	100	0.08	0.49	100
AS precipitation	15	1.82	2.56	87.63	15	0.51	2.23	92.71
Dialysis	15	1.85	2.91	89.37	15	0.52	2.38	93.61
DEAE-Cellulose	6	2.66	4.89	51.28	6	0.79	4.39	56.89
Sephadex G100	4	3.20	5.64	41.80	4	0.85	5.51	40.72

The purity level of the extracted and purified PBPs was different depending on algal species and degree of purification. The purity ratio of the extracted PBPs is an essential property for specific uses (Table 1). According to Rito-Palomares et al.^19^, phycocyanin preparations with an A620/A280 ratio equal to or greater than 0.7 are considered food grade, those with an A620/A280 ratio between 0.7 and 3.9 are reactive grade, and those with an A620/A280 greater than 4.0 are analytical grade and pharmaceutical grade, are sufficient to meet the needs of a specific application. The purity level of all samples was recommended for a specific application, except the crude APC (0.49%), which makes it suitable as an additive source for healthy food^20,21^. The previously studied demonstrated A620/A280 ratio of pure C-PC for *A. platensis* were 5.59, 6.69, and 4.98^22^.

**Figure 4:**
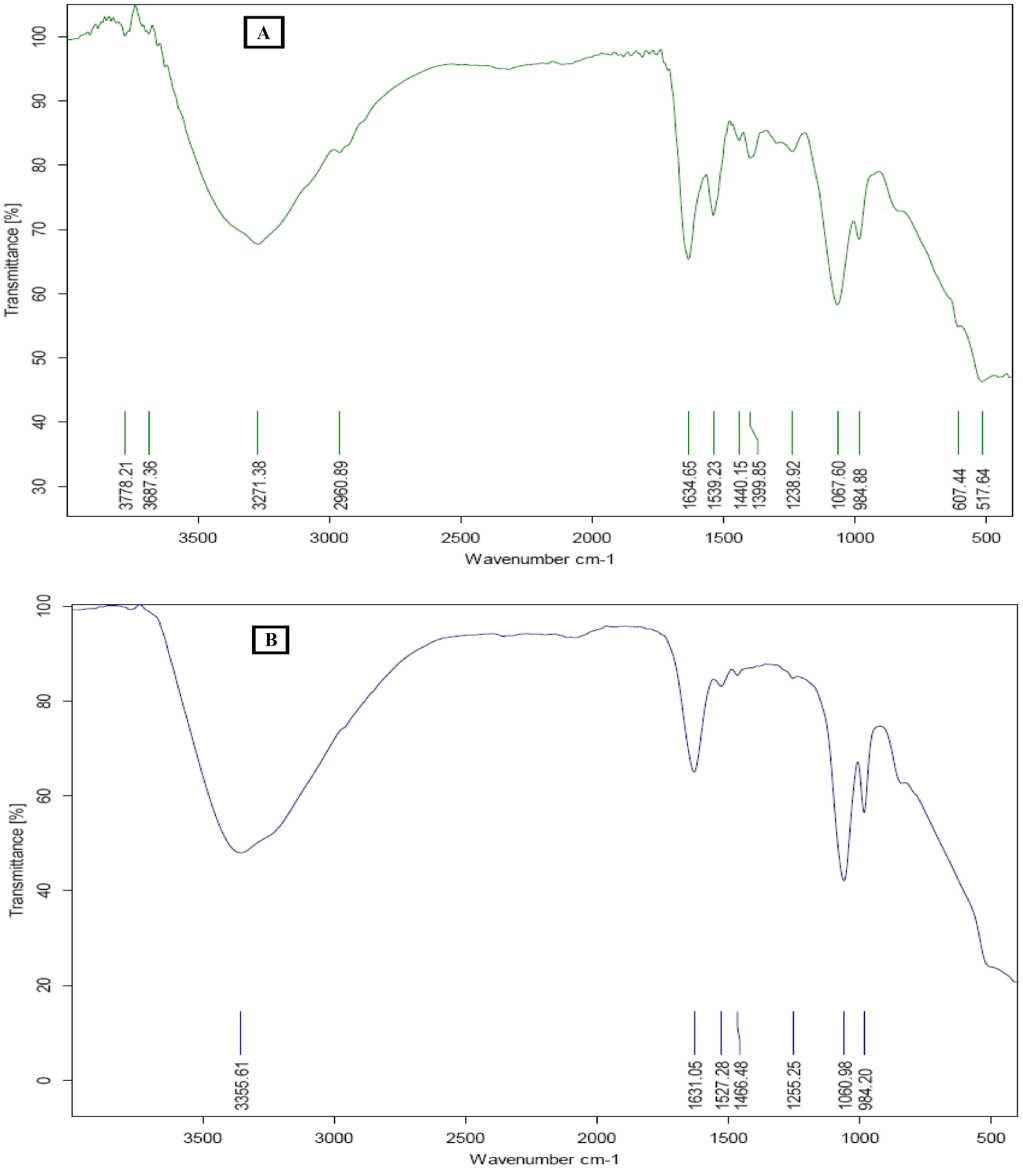
FTIR Spectrum of (**A**) phycocyanin purified from *A. Platensis* and (**B**) allophycocyanin purified from *C. officinalis*.

Figure 4 shows the FT-IR analysis of the purified C-PC of *A. platensis* (Fig. 4A) and the purified APC of *C. officinalis* (Fig. 4B) and demonstrate the protein specific amide I band at 1635/cm for C-PC and at 1631/cm (C=O stretching) and amide II at 1540/cm for C-PC and 1528/cm for APC. Our results are in agreement with the previous studies that showed the proteins amide I and amide II demonstrated vibration bands at 1650/cm and 1645/cm, respectively^23,24^. The position and shape of the amide I band are used to study the secondary structure of proteins. The sharp amide I band for both C-PC and APC reflects the a-helix as the common element of their secondary conformational structure. In addition to this, the FTIR analysis of the purified C-PC and APC further confirmed their purity by the absence of phosphates and inorganic sulfates (representing intense bands at 985/cm and 1061/cm).

### Biological activities

Phycobiliproteins are gaining popularity due to their highly preserved structural and physicochemical characteristics and their potential as an anti-oxidant, anti-inflammatory agents, and immune-stimulatory properties^5^.

### Anti-oxidant activity

**Figure 5:**
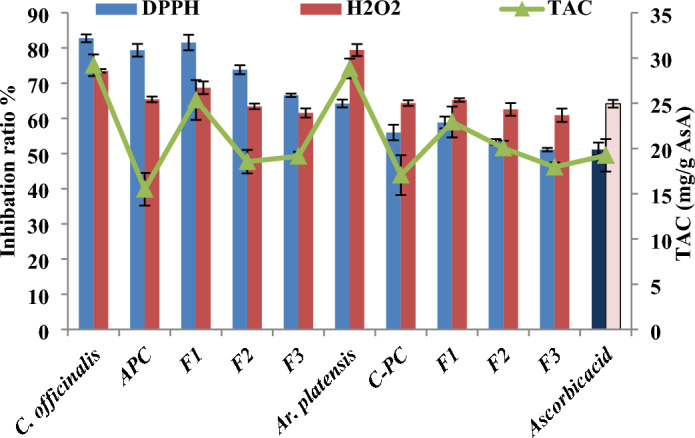
Anti-oxidant activity of the *A. platensis* C-PC, and *C. officinalis* APC and their fractions using different assays.

Anti-oxidant ability of both algal species, the extracted C-PC and APC and their fractions was assessed in vitro using three techniques, as illustrated in Fig. 5. Generally, the anti-oxidant activity of the algal methanolic extracts was higher than that of the purified and fractionated C-PC and APC extracts in all the tested assays. This variation may be due to the fact that both tested species contain different bioactive compounds like polysaccharides, phenolic compounds and fatty acids^21,25^.

There is significant variation in the percent scavenging of DPPH radical by purified and fractionated C-PC and APC from both algal species which, indicating that the tested samples were electron donors and could react with the free radicals and convert them into more stable compounds to terminate the radical chain interaction. PBPs are a natural free radical scavenger, as revealed by numerous studies^26–28^ depending on both primary and secondary pathways by chelating the metal ion that generates ROS^28^. Moreover, the amount and location of the amino acids affect the PBPs’ ability to act as anti-oxidant^11^.

Fraction 1 (F1) exhibited higher scavenging activity than crude, and other two fractions, and standard (ascorbic acid) in the tested species. The results exhibited that the F1 which was purified by ammonium sulfate precipitation extracts, might have some compounds with better DPPH and H_2_O_2_ scavenging. The IC_50_ values of *A. platensis* and *C. officinalis* methanolic extract (604.20 and 778.61 µg/mL), the crude C-PC and ACP methanolic extracts (629.94 and 893.39 µg/mL), and the F1 (613.15 and 850.0 µg/mL) were lower than IC_50_ of ascorbic acid 974.39 µg/mL. This was also reflected in the evaluated IC_50_ values, which were highly significant differences from those recorded for the standard drug (ascorbic acid) (974.39 µg/mL).

Both algal species and their phycobiliproteins samples exhibited comparable total anti-oxidant capacities. The detected data was consistent with that of Sonani et al.^27^ who stated the anti-oxidant ability of PC, PE, and APC from marine cyanobacterium *Lyngbya* sp. A09DM was contributed equally by both chelating activity and reducing activity. Moreover, various cyanobacterial species generated various phycobiliproteins extracts with different anti-oxidant efficiency^29^. Cherdkiatikul and Suwanwong^11^ detected that APC had greater peroxyl radical scavenging activity than C-PC, whereas C-PC had greater hydroxyl radical scavenging activity than APC.

### Anti-inflammatory activity

Recently, anti-inflammatory drugs made from natural marine-based substances have received a lot of attention^12^. Lipoxygenase enzymes catalyse the conversion of arachidonic acid to hydroperoxy eicosatetraenoic acids (HPETEs), which are then reduced to mono-hydroxy eicosatetraenoic acids (mono-HETEs) or (diHETEs), and leukotrienes; these are ranked as one of the most powerful natural mediators of hypersensitivity and inflammation^30^.

As shown in Table 2, the algal methanolic extracts and the C-PC and APC crude and their fractions from each species showed anti-inflammatory activity. The percent inhibitory activity against 15-lipoxygenase by the algal methanol extract and crude extracted PBPs was higher than the purified fractions comparing to ascorbic acid. The selective anti-inflammatory activity of the extracted pigments might be due to many factors, including reducing lipopolysaccharide (LPS) levels, inhibiting the expression of NF-κB, suppressing apoptosis, reducing the autoimmune response, inhibiting cyclooxygenase (COX-2) activity, myeloperoxidase activity, and thus activating macrophages^31^. Moreover, the scavenging properties of PBPs towards oxygen reactive species and their inhibitory effects on COX-2 activity may be the causes of their anti-inflammatory actions^32^. In this context, Ferreira et al.^33^ demonstrated the role of C-PC in anti-inflammatory and anti-oxidant action. C-PC, a substance isolated from *A. platensis*, has been shown to have anti-inflammatory and anti-oxidant effects^34^. The variation in anti-inflammatory activity may be related to the effect of the purification process on the nature of phycobiliproteins which are proteins in nature and are affected by a variety of factors, such as extraction process, temperature, chemical types, and ratio^29^.

**Table 2:** Anti-inflammatory and anti-arthritic activities(%) of the selected algae and their purified CPs.

Algal spp.	Activity %	
	Anti-inflammatory activity %	Anti-arthritic activity %
*C. officinalis*	74.546^a^	87.5^a^
Crude APC	74.813^a^	78.25^b^
F1	75.987^a^	73.89^c^
F2	74.013^a^	74.25^c^
F3	62.520^c^	68.54^d^
*Ar. platensis*	75.347^a^	86.32^a^
Crude C-PC	74.493^a^	76.98^b^
F1	74.546^a^	75.02^b^
F2	71.451^b^	73.21^a^
F3	67.820^b^	69.54^b^
Diclofenac sodium	72.020^b^	71.5^b^

In addition, the estimated IC_50_ values were 670.73, 668.33, 658.01, 675.56 and 799.74 µg/mL for *C. officinalis*, crude APC, F1, F2, and F3*,* respectively, likened to Diclofenac at 689.46 µg/mL. While the evaluated IC_50_ values for *A. platensis* methanolic extract, C-PC, F1, F2, and F3 were 663.6, 671.2, 670.73, 699.78 and 737.25 µg/mL. These results indicated that anti-inflammatory activity not only differs according to the algal species, but also according to the structure of the extracted phycocyanin and its fractions.

#### Antiarthritic activity

With respect to the anti-denaturation activity of the tested algae and their extracted phycobiliproteins fractions (Table 2), the anti-denaturation ability was used to test their ability to control the production of autoantigens and thereby inhibit the denaturation of proteins as compared to diclofenac sodium as a standard drug, where the major cause of rheumatoid arthritis is the denaturation of proteins and production of autoantigens^35^. In contrast to the medication diclofenac sodium, the samples demonstrated their potential to limit the synthesis of autoantigens and consequently inhibit the denaturation of proteins, which is a key contributing factor to rheumatoid arthritis^35^. It was apparent that the methanolic algal extracts and crude C-PC and APC of both tested species had higher anti-arthritic activity than fractions. *C. officinalis* and *A. platensis* methanolic extracts contained different bioactive substances which had the anti-denaturation activity^21,36^.

The evaluated IC_50_ were in the following order *C. officinalis* methanolic extract (571.43 µg/mL), *A. platensis* (579.24 µg/mL), C-PC (638.98 µg/mL), and APC (649.52 µg/mL), which were significantly compared to diclofenac value (699.3 µg/mL). The antiarthritic effects of PBPs may be due to their ability to scavenge ROS as well as their efficiency in blocking the metabolism of arachidonic acid and the generation of cytokines like tumor necrosis factor (TNF)^37^.

#### Anti-bacterial activity

In this study, we investigated the antimicrobial effect of the tested algae and their PBPs fractions against marine pathogenic bacteria by turbidity measurement under sterile laboratory conditions. It was found that the methanolic algal extracts and crude PBPs of both tested species had higher antibacterial activity than PBPs fractions (Table 3). Whereas *C. officinalis* and *A. platensis* methanolic extracts contained different bioactive substances which inhibit the bacterial growth at different levels depending on the source of the extract, the extraction solvent, and the concentration of the pigment in the extract, as well as the tested marine pathogens as demonstrated in previous studies^36,38^. The anti-microbial activity of PBPs was depended on protein nature of these pigments and hydrophobic properties of their amino acids content^39^. The results showed that methanolic algae extracts and crude PBPs of the two tested species exhibited higher anti-bacterial activity against Gram +ve marine bacteria than Gram −ve species. The resistance of the Gram −ve bacteria may be due to their outer membrane structure, or due to the presence of resistance genes and DNA from other resistance strains, or genetic changes in the DNA which can change protein production and lead to receptors that recognize the antibiotics^40^. In this context Gentscheva^41^ indicated that the methanol, ethanol and aqueous extracts of the *A. platensis* have antimicrobial activity against four different types of Gram +ve bacteria, namely, *S. aureus, S. pneumonia*, *B. cereus, E. faecalis*, no antimicrobial activity for the methanolic extract give positive results with *S. pneumonia*, and *B. cereus.* Also, Kovaleski^42^ reported the anti-microbial activity of the extracted PBPs from red algae, and Safari^4^ recorded that the inhibitory activity of C-PC from *A. platensis* on *S. aureus*.

**Table 3:** Anti-bacterial activity of the tested algae and their purified CPs.

Marine pathogenic strains	Type of organisms	Inhibition activity %										
		*C. officinalis*					*Ar. spirulina*					Positive control
		Algal extract	Crude CP	F1	F2	F3	Algal extract	Crude CP	F1	F2	F3	
*S. aureus*	Gram +ve	0	33.7	27.4	15.3	34	36.9	21.2	0	5.5	28.8	33.6
*B. subtilis*	Gram +ve	31.3	32.6	25.9	14.5	8.8	13.6	24.4	11.1	24	17.6	17.2
*E*.* faecalis*	Gram +ve	26.5	31.5	18.6	12.1	10.3	17.7	20.6	5.4	16.5	12.3	20.5
*S. agalactiae*	Gram +ve	33.1	33.7	31.5	29.6	22.2	20.8	31.1	22.8	30.8	42.7	15.5
*P. aeruginosa*	Gram −ve	0	28.9	15.4	5	5.1	18	0.2	4.8	24.3	15	48.7
*E.coli*	Gram −ve	23.1	3.5	22.1	16.4	11.2	16.5	20.5	22.2	12.4	18.6	51.7
*K. pneumonia*	Gram −ve	28.5	43.8	27.6	23.7	24.9	29.0	30.7	31.7	21.2	24.3	57.5
*V. damsela*	Gram −ve	34.6	19.6	19.9	23.7	16.8	34.6	12.4	20.4	23.4	14.1	24.9
*V. fluvialis*	Gram −ve	19.3	9.3	14.7	10.5	16.4	0	14.2	17.5	11.5	14.7	25.6
*P. fluorescence*	Gram −ve	3.2	9.6	10.6	6.1	7.1	0	10.0	10.9	24.3	21.6	23.9
*A. hydrophila*	Gram −ve	9.2	0	2.5	2.2	11.4	10.4	13	0	5.2	9.6	10.8

## Conclusion

Red algae and cyanobacteria's phycobiliproteins are proteinaceous pigment valuable byproducts with a variety of economical applications. Recently, it has become more crucial to replace the synthetic colours due to their beneficial effects. The results of the present study clearly demonstrate that the crude C-PC and APC and their fractions from *A. platensis*, and *C. officinalis* exhibited high biological activities in vitro, including anti-oxidant, anti-inflammatory activity, anti-arthritic, and anti-microbial activities, with a different ratios depending on the algal species, and purification degree. These findings provide the pigment's reference data, which can be used in the food, drug, and cosmetic industries. Future studies should be directed towards improving the PBP production and also improve their biological activities.

## Materials and methods

### The tested algae

#### Microalgal culture

*Arthrospira* (*Spirulina*) *platensis* (Geitler) from Cyanophyceae was isolated from the Eastern Harbor seawater, Alexandria, Egypt, then isolated and cultured in the laboratory on Zarrouk medium^43^. Identification of the species was done according to Desikachary^44^ and the Algae Base website^45^. The culture of *A. platensis* was incubated at 30 °C under 12:12/light–dark cycles at 40 μE/m^2^/s light intensity. Then it was harvested in the stationary stage (after 12 days) by centrifugation at 4500 × g for 20 min and the obtained biomass was washed thoroughly using deionized water to remove adhering salts. Finally, the pellet was freeze-dried to a constant weight at 60 °C, and then stored in a glass vial in a refrigerator at − 20 °C for further analysis.

#### Macroalgal sample

Fresh *Corallina officinalis* Linnaeus from Rhodophyceae was handpicked during the summer season 2021 from the rocky offshore distinct along the Eastern Harbor (longitudes 29.88°–29.90° E and latitudes 31.20°–31.22° N). The collected sample was washed with distilled water and then cleaned with a soft brush to remove epiphytes and extraneous substances. On the same day of collection, some of the collected seaweeds were preserved in formalin (5%) in seawater for taxonomical identification according to Aleem^46^, then confirmed with the Algae Base website^45^. Some of the clean thallus was dried to a constant weight at room temperature (27 ± 2 °C) on absorbent paper, then ground into fine powder and stored at − 20 °C for further uses.

#### Phycobiliproteins extraction, purification, and characterization

About one gram from each dried biomass was resuspended in 0.1 M phosphate buffer, pH 6.8, as a working buffer (WB) and both suspended biomasses were disrupted by sonication for 60 s. Then, extraction of *A. platensis* and *C. officinalis* phycobiliproteins was performed in WB by repeated freezing at – 20 °C and thawing at room temperature in the dark condition once daily for 4 days until cell extracts became dark blue. Both *A. platensis* and *C. officinalis* phycobiliproteins mixtures were subsequently centrifuged at 10,000 × g for 30 min at 4 °C and clear supernatant was collected. The absorbance of each extraction was measured at 562, 620, and 652 nm against WB as a blank reference for calculating the concentrations of C-phycocyanin (C-PC), allophycocyanin (APC), and phycoerythrin (PE)^47,48^. For the purification of both *A. platensis* and *C. officinalis* phycobiliproteins, the crude extract of each biomass was precipitated by adding 65% ammonium sulphate slowly through continuous stirring for achieving 65% saturation with ammonium sulphate. After standing each solution in a cold room for 12 h, each solution was centrifuged at 4500 × g for 10 min and each pellet was resuspended in the small volume of WB and dialyzed for 3 days against ten times the volume of WB^49^. The obtained dialyzed solutions were applied separately to DEAE–cellulose column pre-equilibrated with WB and eluted with acetate buffer with pH ranging from 3.8 to 5.6 to develop the column at the flow rate of 20 mL/h^49^. All fractions containing phycobiliproteins were collected and concentrated by ultrafiltration with cut off 3 KDa (Millipore, Merk, USA) and applied to a Sephadex G100 column equilibrated with WB. Phycobiliprotein for each *A. platensis* and *C. officinalis* phycobiliproteins were eluted from a Sephadex G100 column at a flow rate of 0.5 mL/min using WB containing 0.15 M NaCl. Both purified red algae and cyanobacterial phycobiliproteins were characterized during each purification step and analysed by absorption spectroscopy via scanning the fractions in a range of 260–750 nm. Purity of each phycobiliproteins from *A. platensis* and *C. officinalis* was calculated at each purification step according to the absorbance ratios of A620/A280 and A650/A280 for C-PC and APC, respectively^50^. The purity and homogeneity of each algal C-PC and APC were analysed by 12% SDS-PAGE with medium range protein ladder (15–240 Mw). All fractions contain the purified C-PC or APC were pooled, dialyzed, lyophilized, and kept in − 20 °C until further uses. In addition, the purified C-PC and APC were characterized by FTIR (Shimadzu FT-IR-8400 S, Japan).

### Biological activities of the tested algae and the purified C-PC and APC

#### Anti-oxidant activity

The anti-oxidant activity of the selected algae and the extracted phycobiliproteins (1000 µg/mL) was determined in methanolic extracts using three standard methods.

#### 1, 1-Diphenyl-2-picrylhydrazyl (*DPPH*) radical scavenging activity assay

The ability of the tested algae and their phycocyanin extracts to scavenge DPPH free radicals was estimated according to Tierney et al.^51^ technique. The DPPH scavenging activity which was scavenged was calculated using the following formula:

$${\text{DPPH}}\;\;Scavenging \,activity \% = \left[ {1 - \frac{{{\text{AC}} - {\text{AS}}}}{{{\text{AC}}}}} \right] \times 100$$

where AC is the absorbance of the control, and AS is the absorbance in the presence of the sample or standard.

Concentrations of 50% inhibition (IC_50_) of DPPH radicals were calculated by GraphPad Prism 6 software.

#### The total anti-oxidant capacity (TAC)

TAC of the different samples was analyzed by spectrophotometry at 695 nm. However, the detected TAC was equivalent to the ascorbic acid standard (0.5 g/100 mL distilled water) and was expressed as mg/g ascorbic acid equivalent (AsA equivalent) according to the Prieto et al.^52^ method.

#### Hydrogen peroxide radical scavenging ability

H_2_O_2_ scavenging activity of the tested samples and or ascorbic acid as the control was determined at 230 nm according to Gülçin et al.^53^.

The percentage of H_2_O_2_ scavenging activity was estimated using the formula:

$${\text{H}}_{{2}} {\text{O}}_{{2}} \;Scavenging \;activity \; \% = \left[ {\frac{{{\text{[AC}} - {\text{AS]}}}}{{{\text{AC}}}}} \right] \times 100$$

The IC_50_ values were also calculated as described above.

#### Anti‑inflammatory efficiency (15‑lipoxygenase inhibitory assay)

The anti-lipoxygenase activity assay of the sample methanolic extracts was based on measuring the formation of the complex Fe3+/xylenol orange using a spectrophotometer at 560 nm^54^. The inhibition ratio was calculated using the following equation using quercetin “standard drug” as a standard drug.

$${\text{Anti-inflammatory activity }}\; = \;\frac{{\left( {\left( {A \;{\text{control }} - { }A \;{\text{blank}}} \right){ } - { }\left( {A \,{\text{sample }} - { }A \;{\text{blank}}} \right)} \right)}}{{\left( {A\; {\text{control }} - { }A\; {\text{blank}}} \right){ }}} \times 100$$

where *A* control is the absorbance of control, *A* blank is the absorbance of blank, and *A* sample is the absorbance of the sample.

#### Anti‑arthritic activity (protein denaturation assay)

Anti-denaturation ability of the tested samples was done by Sakat et al.^55^ method with slight modifications. Diclofenac sodium “commercial drug” and distilled water were used as positive and negative controls, respectively. The inhibition percentage was measured at 660 nm and estimated using the following formula.

$${\text{Anti-arthritic activity \%}}\; = \;\frac{{\left ( {\left({A\; {\text{sample}} - { } A\; {\text{blank}}} \right)} \right)}}{{\left ( {A \; {\text{control}}} \right) { }}}\times 100$$

where *A* control is the absorbance of control, *A* blank is the absorbance of blank, and *A* sample is the absorbance of the sample.

### Anti-bacterial assay

#### Pathogenic bacteria

The tested pathogenic gram negative bacterial strains “*Escherichia coli* ATCC8739*, Vibrio fluvialis* ATCC33809, *V. fluvialis* ATCC33809, *Pseudomonas aeruginosa* ATCC9027, *P. fluorescence* ATCC13525, *Klebsiella pneumonia* ATCC13883, and *Aeromonas hydrophila* ATCC13037”, and the gram positive *Bacillus sutlils* ATCC6633, *Enterococcus faecalis* ATCC29212, *Streptococcus agalactiae* ATCC13813, and *Staphylococcus aureus* ATCC25923 were obtained from the Marine Microbiology Laboratory, NIOF, Alexandria.

#### Preparation of bacterial culture

Using aseptic techniques, bacterial single colony was transferred into a 100 mL nutrient broth and placed in an incubator overnight at temperature 37 °C. After 24 h of incubation, the bacterial broth culture was centrifuged at 6000 rpm for 15 min, then a clean pellet of bacteria was prepared. The broth was spun down using suitable aseptic precautions. The resulted supernatant was discarded into a labeled waste beaker. The resulted pellet was re-suspended in 20 mL of sterile saline solution and centrifuged again at 6000 rpm for 15 min. This step was repeated many times until the obtained supernatant became clear. Then the pellet was suspended in 20 mL of sterile saline, and labeled as Bs (Broth suspension). The Bs optical density was recorded at wave range 600 nm, and serial dilutions were carried out with suitable aseptic methods until the Bs optical density reached the range of 0.5–1.0. The actual colony-forming units’ number (CFU) was calculated and estimated from the graph of viability. The needed dilution factor was estimated and the serial dilution was carried out to reach a concentration of 5 × 10^6^ cfu/mL^56^.

### The bacterial inhibition assay

Labelled test tubes were prepared under aseptic conditions. 10 μL of the test algal extract dissolved in 1000 µg/mL (w/v) DMSO stock (usually a stock concentration of 1 mg/mL for purified compounds), antibiotic (as a positive control) and nutrient broth only (as a negative control) were pipetted into 900 μL nutrient broth containing test tubes. Finally, 10 μL of bacterial suspension was added to each test tube to reach a concentration of 5 × 10^6^ cfu/mL. Then the obtained test tubes were wrapped loosely with cling film. Each rack had test tubes with a set of controls, broad-spectrum antibiotic as a positive control (usually ciprofloxacin in the same concentration as the test compound for bacteria and diclofan for the yeast), all solutions with the exception of the test compound, and all solutions with the exception of the bacterial solution adding 10 μL of nutrient broth^56^.

### Use of standardized bacterial colony numbers

According to Macfarland standards method, usually 0.5 turbidity is not able to give a CFU standard number for all bacterial strains so it is very difficult to compare between different bacterial species due to optical densities differences. So to obtain a uniform bacterial number of different species a set of killing/viability graphs for each bacterial species must be prepared. A final concentration reach 5 × 10^6^ cfu/Ml. Thus, different bacterial species and different strains could be compared^57^.

### Statistical analysis

The obtained results were expressed as standard deviation mean ± (n = 3) and the differences statistical significance between the different treatments was estimated using one-way analysis of variance (ANOVA) using the program SPSS 15.0 statistical software. Differences were accepted and considered significant at P value < 0.05.

## Data Availability

All data produced during this study are included in this published article.
